# A Public Health Approach to Automated Pain Intensity Recognition in Chest Pain Patients via Facial Expression Analysis for Emergency Care Prioritization

**DOI:** 10.3390/diagnostics15202661

**Published:** 2025-10-21

**Authors:** Rita Wiryasaputra, Yu-Tse Tsan, Qi-Xiang Zhang, Hsing-Hung Liu, Yu-Wei Chan, Chao-Tung Yang

**Affiliations:** 1 Department of Industrial Engineering and Enterprise Information, Tunghai University, Taichung 407224, Taiwan; rita.wiryasaputra@ukrida.ac.id; 2 Informatics Department, Krida Wacana Christian University, Jakarta 11470, Indonesia; 3 School of Medicine, Chung Shan Medical University, Taichung 402306, Taiwan; janyuhjer@gmail.com; 4 Department of Emergency Medicine, Institute of Occupational Medicine, Taichung Veterans General Hospital, Taichung 407219, Taiwan; ph0422ph0422@gmail.com; 5 Department of Occupational Safety and Health Office, Taichung Veterans General Hospital, Taichung 407219, Taiwan; 6 Department of Post-Baccalaureate Medicine, College of Medicine, National Chung Hsing University, Taichung 407224, Taiwan; 7 Institute of Computer Science and Engineering, National Yang Ming Chiao Tung University, Hsinchu 300093, Taiwan; 8 Department of Emergency Medicine, Kuang Tien General Hospital, Taichung 433004, Taiwan; 9 Bachelor Degree Program of Artificial Intelligence, National Taichung University of Science and Technology, Taichung 404336, Taiwan; ywchan1103@nutc.edu.tw; 10 Department of Computer Science, Tunghai University, Taichung 407224, Taiwan; 11 Research Center for Smart Sustainable Circular Economy, Tunghai University, Taichung 407224, Taiwan; 12 Department of Medical Research, Kuang Tien General Hospital, Taichung 433004, Taiwan

**Keywords:** cardiovascular disease, chest pain intensity, deep learning, emergency triage, facial expression, expression recognition, YOLO

## Abstract

**Background/Objectives**: Cardiovascular disease remains a leading cause of death worldwide, with chest pain often serving as an initial reason for emergency visits. However, the severity of chest pain does not necessarily correlate with the severity of myocardial infarction. Facial expressions are an essential medium to convey the intensity of pain, particularly in patients experiencing speech difficulties. Automating the recognition of facial pain expression may therefore provide an auxiliary tool for monitoring chest pain without replacing clinical diagnosis. **Methods**: Using streaming technology, the system captures real-time facial expressions and classifies pain levels using a deep learning framework. The PSPI scores were incorporated with the YOLO models to ensure precise classification. Through extensive fine-tuning, we compare the performance of YOLO-series models, evaluating both computational efficiency and diagnostic accuracy rather than focusing solely on accuracy or processing time. **Results**: The custom YOLOv4 model demonstrated superior performance in pain level recognition, achieving a precision of 97% and the fastest training time. The system integrates a web-based interface with color-coded pain indicators, which can be deployed on smartphones and laptops for flexible use in healthcare settings. **Conclusions**: This study demonstrates the potential of automating pain assessment based on facial expressions to assist healthcare professionals in observing patient discomfort. Importantly, the approach does not infer the underlying cause of myocardial infarction. Future work will incorporate clinical metadata and a lightweight edge computing model to enable real-time pain monitoring in diverse care environments, which may support patient monitoring and assist in clinical observation.

## 1. Introduction

### 1.1. Background Study

Cardiovascular disease (CVD) is a general term that encompassess coronary heart disease, rheumatic heart disease, peripheral arterial disease, heart failure, congenital heart disease, cerebrovascular disease (stroke), and elevated blood pressure (hypertension). CVD is the most frequent cause of mortality globally with an estimated 18.6 million deaths annually [1,2]. It has been emphasized that climate change, lifestyle choices, and genetic factors are factors of its further development, making CVD one of the most urgent issues of general health in the population [3]. The World Health Organization (WHO) estimates that four in five deaths related to CVD are attributed to heart attacks and strokes, and more importantly, a third of these deaths happen before the age of 70 years [4].

The human face serves as a primary information resource [5], and the manifestation of chest pain is reflected in facial expressions as it is one of the most efficient means of conveying pain information [2,6,7]. Moreover, facial expressions constitute the most reliable indicator for assessing human pain emotions that apply to any healthcare environment [8]. Many previous works have applied the Facial Action Coding System (FACS) method that transforms every observable facial expression into a set of component movements [9,10,11,12]. The FACS method works as a model-based approach to obtaining facial features through frame-by-frame scanning [8] and provides values for valence and arousal levels as well as pain severity assessments of distinct facial expressions. The most obvious pain severity factor exists at varying levels of intensity from low to moderate to high. The pain level is measured using the Prkachin and Solomon Pain Intensity (PSPI) that employs the FACS to assess pain through facial muscle identification, which defines Action Units (AUs) [13].

The advancement of cutting-edge technology and machine learning brings a new horizon in identifying pain levels through facial recognition, deduced from the movements in facial muscles and their correspondence with PSPI scores. Typically, the Convolutional Neural Network (CNN) framework, as a successful computational model used in machine learning, is employed for facial recognition tasks [14]. In real-time efficiency, the CNN approach, the main foundation of the You Only Look Once (YOLO) object detection framework, can be utilized in facial recognition even though it is uncommon. YOLO’s primary strength as a single-phase object detector lies in its ability to simultaneously localize and classify objects within images or videos in real time [14,15]. This efficiency—combined with high accuracy and low computational cost [16]—makes it particularly suitable for clinical pain assessment, where rapid analysis of facial expressions is critical. Hasan revealed that human facial emotion recognition is a supporting medium for medical personnel in personalizing effective care. His research, using the YOLOv3 model, was able to classify facial expressions into seven emotions, achieving an accuracy of 94% on the FER2013 dataset [17]. Their proposed model demonstrates both robustness and speed in emotion classification, including in identifying items in an image or video stream. Not only has the development of the YOLO approach become a constant interest to researchers, but research related to face detection has expanded to include masked face detection. As the successor to the YOLOv3, Han [18] proved that their YOLOv4-tiny modified approach was superior in face recognition, particularly in the scope of mask-wearing detection from the YOLOv4-tiny baseline; their F1-scores reached 55%. As such, the journey of future advancements using YOLO offers numerous exciting possibilities. However, only a few studies have focused on the level of pain intensity accompanied by the YOLO model. These studies not only focus on human objects but also animal objects. Vidal [19] used the YOLOv3 framework for the automation of face detection that supports the pain scale of mice. Their recall and precision rates achieved excellent values at 100%, indicating that the YOLO model has very high performance. By harnessing YOLO’s inherent object detection and localization capabilities, our previous work [20] focused primarily on the overall performance of YOLOv4, YOLOv6, and YOLOv7 for detecting facial expressions in patients experiencing chest pain. YOLOv4 and YOLOv6 achieved 80–100% accuracy in detecting pain through facial expressions, outperforming YOLOv7. However, methods for distinguishing the level of pain intensity have not been discussed in the study.

To summarize, there is still a gap between pain assessment and the utilization of the YOLO approach in the ER, although the approach ultimately supports emergency treatments and improves patient care outcomes. This study proposes a web-based chronic non-communicable disease pain intensity level automation system (PILASFE) that utilizes streaming technology for capturing facial expressions in patients experiencing chest pain. The system is intended to assist medical staff in emergency settings by allowing for real-time objective monitoring of pain intensity as a complementary decision support tool. Therefore, medical professionals may be better supported in monitoring patients and making timely clinical observations. With the aim of improving the life quality for patients with chronic CVD, the key contributions of this study are as follows:

- Development of a facial expression-based pain recognition system by fine-tuning YOLO models with PSPI scores, enabling accurate detection and visualization of pain levels in real-time;
- Integration of streaming technology to ensure uninterrupted video input for consistent facial expression monitoring and implementation of a web-based interface with color-coded indicators, enhancing accessibility and usability;
- Provision of a decision support tool with potential future applications in emergency care settings where healthcare personnel are limited.

The abbreviations that are used in the manuscript are shown in Abbreviations part. The sections are organized as follows: the materials and research methodology are presented in Section 2, while Section 3 and Section 4 cover the experimental results and discussion. Finally, the conclusion and future work are presented in Section 5.

### 1.2. Literature Review

To provide a comprehensive overview of pain assessment, recent advancements in CNN architectures have demonstrated growing efficacy in facial recognition tasks. The continous evolution of these architectures highlights their potential for improving accuracy, robustness, and clinical applicability. Table 1 presents previous studies on pain assessment, outlining diverse methodologies, datasets, and performance outcomes. Chavan [21] aimed to enhance accuracy in pain recognition through a hybrid optimization-based deep CNN framework. By leveraging ResNet101 for feature extraction, their model achieved high accuracy of 94.95%, sensitivity of 97.33%, and specificity of 99.04%. However, it suffered from vanishing gradients and overfitting due to dataset imbalance. Similarly, Ismail [22] focused on improving patient-centered pain management in the United Arab Emirates by proposing a deep learning framework that addressed class imbalance through weighted loss functions and data augmentation. Their ResNet18 model outperformed VGG-Face and ResNet34, achieving an accuracy of 87.89% in 5-fold cross validation. Hausmann [23] targeted timely pain management in post-surgical neonates in the Neonatal Intensive Care Unit by employing YOLOv3, YOLOv5, and YOLOv6. Their results showed that YOLOv6 outperformed other models, achieving a precision of 63.2% and an accuracy of 62.7%, though generalization was limited because the models were trained primarily on an adult dataset.

Gkikas [24] conducted a study on multimodal automatic acute pain assessment using a big framework with fewer than 10 million total parameters. Their proposed framework was divided into several modules and employed six datasets: the VGGFace dataset, AffectNet, Compound Facial Expressions of Emotions Database, RAF Face Database basic, RAF Face Database compound, and the ECG Heartbeat Categorization dataset. A multitask cascaded convolutional neural network detector was applied for face and landmark prediction. In the binary pain classification task, the model achieved an accuracy of 82.74%. Although its performance in the multi-level pain classification task was considerably lower, with an accuracy of only 39.77%, this result was still an improvement compared to previous studies on similar tasks. The complexity of the framework contributed to improving interpretability, which may support its clinical acceptance. However, this complexity also introduced drawbacks, particularly an increase in inference time due to the pipeline approach. Swetha [25] conducted a study of automatic pain detection based on the extracted images from the pain expression database using the Dense-CNN architecture. The images of facial expressions were collected by themselves and were classified into multi-class classifications, namely no pain, moderate pain, intolerable pain, and severe pain. The performance of their proposed model achieved an accuracy of 75%. By using facial images, Wu [26] identified four pain intensity levels—no pain, weak pain, mild pain, and strong pain—based on the CNN approach with the combination of local and global features of the GANet module and LANet module. The model was validated in 5-fold cross validation and achieved its accuracy at 56.45%. Based on the images from the videos, Alghamdi revealed that the reliability of the CNN method was able to improve pain management in the multi-classification scenario, with no pain, low pain, moderate pain, and severe pain being detected through facial expressions [13]. Their proposed work utilized two concurrent subsystems that analyze the entire face and the upper half of the face using a CNN. The proposed model’s performance achieved an accuracy of 99.10% on a 10-fold cross validation. Collectively, these studies illustrate diverse objectives: improving classification accuracy, addressing class imbalance, and refining multi-level classification.

## 2. Materials and Methods

This section explains data collection, preprocessing, model implementation, and performance evaluation processes.

### 2.1. Data Collection

The collected data comprised data about patients experiencing symptoms of chest pain in the emergency department of Taichung Veterans General Hospital, patients’ 3-min questionnaires, and the physician’s evaluations in line with the ethical standards and approval of the Institutional Review Board. The medical staff differentiated between patients who reported chest pain syndromes and patients who reported no chest pain. Participants completed an eight-item questionnaire regarding their chest pain conditions and provided written consent for voluntary participation. They were informed that all collected data would be used for research purposes. The study enrolled 182 participants, consisting of 84 women and 98 men, with ages ranging from 13 to 96 years. The average age of the participants was 55 years. The mean triage level score was 2.7, which means moderate urgency, while the average treatment value was 0.8, indicating a low probability of admission to the hospital or critical intervention. For data collection, participants were seated approximately one meter away from the network camera mounted in the ER. The camera was positioned to capture the patients’ facial expressions clearly. Each recording session lasted up to five minutes, with videos captured at 30 frames per second (FPS) with a full HD resolution of 1920 × 1080 pixels. To analyze pain dynamics, video frames were extracted every second and saved as individual images. Due to COVID-19, some participants wore face masks as a precaution against transmission. Thus, pain intensity detection parameters accounted for both masked and unmasked faces. To ensure dataset robustness, the study combined patient-collected data with the UNBC-McMaster Shoulder Pain Archive dataset—a widely used benchmark in pain research [13,26,27,28,29,30,31,32,33].

**Figure 1:**
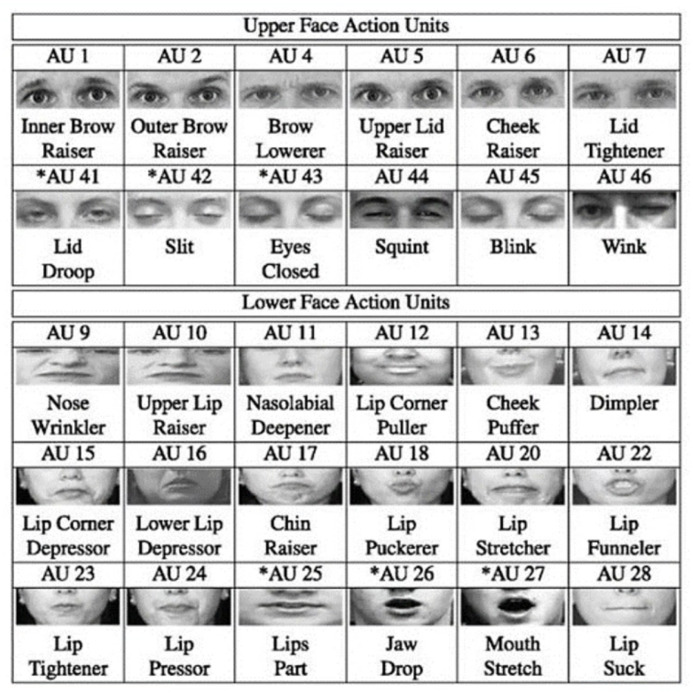
The codes of AUs. Asterisks indicate AUs that are commonly associated with pain-related facial movements.

Pain information is primarily characterized by four core factors [34]. Pain intensity is quantified using the PSPI index, a standardized metric derived from frame-by-frame FACS analysis, which measures specific AUs associated with pain expressions. The unique codes of AUs are shown in Figure 1. The PSPI scale (shown in Equation (1)) represents a discrete ordinal level, ranging from no pain (0) to maximum pain (16) [9,27], with scores categorized into three clinically meaningful levels. Level 1 (no-pain class; PSPI = 0) was characterized by neutral facial expressions. Level 2 (mild pain; PSPI = 1–6) manifested through subtle facial cues, including brow lowering (AU4) and partial eye closure. Level 3 (severe pain; PSPI = 7–16) displayed pronounced distress signals, including full orbital tightening (AU6 + AU7) and prominent levator contraction (AU9). This standardized classification scheme enabled consistent severity assessment across all facial recordings, regardless of masking status, while maintaining compatibility with existing pain research paradigms.

(1) PSPIpain=AU4+max(AU6,AU7)+max(AU9,AU10)+AU43

where [9,27]

- AU4 focuses on brow lowering.
- AU6 shows cheek raising.
- AU7 represents eyelid tightening.
- AU9 represents nose wrinkling.
- AU10 indicates the upper lip raising.
- AU43 represents eye closure.

After obtaining data, the facial expression images were classified into three distinct pain levels based on PSPI scores. The dataset distribution ensures adequate representation of all the pain intensities of the occluded and non-occluded facial expressions. The sample distribution based on pain levels is detailed in Table 2. The distribution number of maskless samples was defined as follows: the level 1 category had 3960 samples, there were 3872 samples in level 2, and 3889 samples were included in level 3. The numbers of mask sample distributions were defined as follows: level 1 had 4627 samples, level 2 had 5627 samples, and 5456 samples were included in level 3.

### 2.2. Data Preprocessing

**Figure 2:**
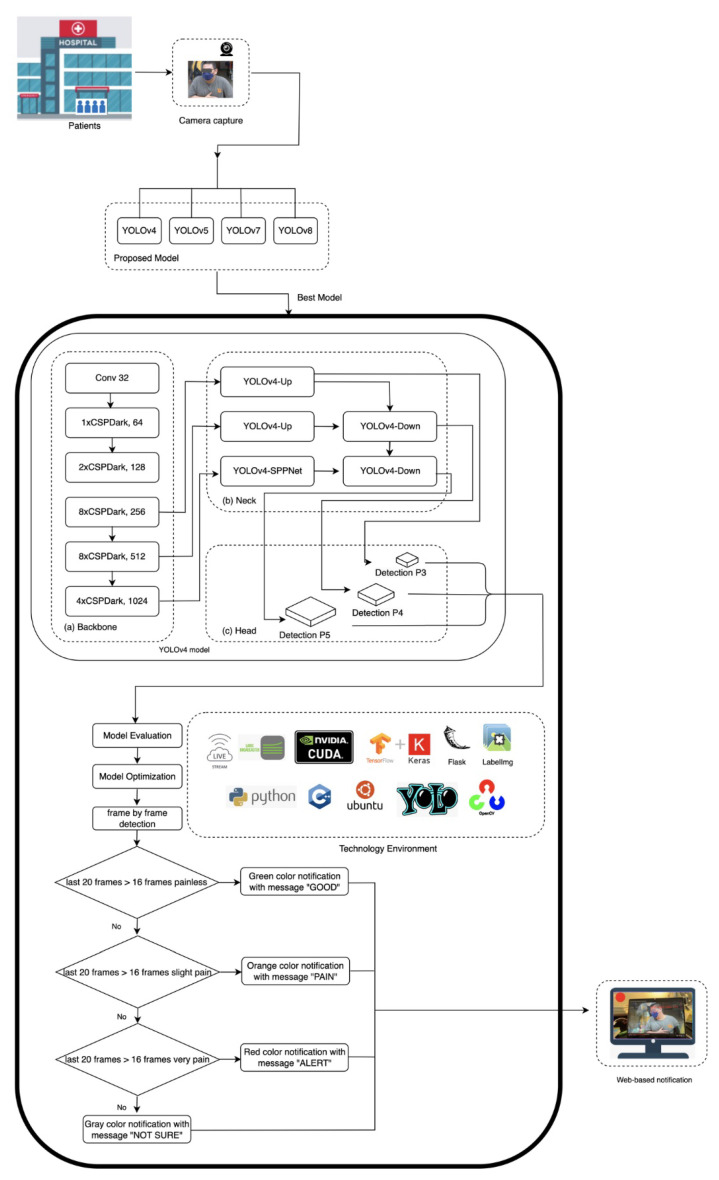
The proposed model architecture.

The proposed architecture is illustrated in Figure 2. The pseudocode object detection model is described in the Supplementary File. In order to make the model more robust, data augmentation techniques including rotation, horizontal flipping, and photometric adjustments were applied to diversify the data while preserving pain-related facial features. All the images have been subsequently labeled with LabelImg to provide multi-class labeling of pain and were used to provide consistent ground truth to train the model. The balanced and expanded dataset allowed us to determine which facial AUs allowed for reliable detection of pain-related facial AUs on the entire spectrum of clinical severity.

### 2.3. Model Development

The main environment uses the operating systems Linux Ubuntu 22.04, language programming Python 3, compute unified device architecture (CUDA), CUDA deep neural network (cuDNN) library, zlib compression library, CMake compatibility platform, OpenCV library, Tensorflow framework, Keras framework, and Darknet framework. The Keras framework preprocessed the data, and then the Tensorflow framework trained the deep learning model. YOLO provides several scaled architecture versions: t (tiny), n (nano), s (small), m (medium), l (large), and x (extra large). The model development pipeline employed an architecture selection between YOLOv4 and YOLOv4t as its lightweight variant for real-time detection of chest pain intensity. While YOLOv4t offered faster inference speeds for real-time ER triage, its accuracy was lower than that of YOLOv4 on benchmark datasets, prompting a comparative evaluation of both architectures. We initialized training using the YOLOv4.conv.137 pre-trained weights for YOLOv4 whilst the YOLOv4-tiny.conv.29 pre-trained weights were used for the YOLOv4t. In the actual tests and evaluations, we found that YOLOv4t was less recognizable. Given the absence of fast-moving subjects in clinical settings and the critical requirement for precise pain intensity assessment, the YOLOv4 model was selected as the base architecture due to its demonstrated superiority in analyzing pain-related facial expression.

Our implementation leverages the Darknet framework, utilizing the parameters detailed in Table 3, to deploy a hierarchical feature extraction pipeline optimized for determining pain-related facial AUs. Since our model employed three classes, the initial batch configuration is set to 6000 according to Equation (2) [35]. As illustrated in Figure 2, our YOLO’s architecture integrates a core structure of backbone, neck, and head components. The first layer of YOLOv4’s network architecture is a 3 × 3 convolutional layer (32 filters), which is used to extract basic facial texture, serving as the foundation for subsequent pain expression analysis. This feeds into a backbone comprising five Cross-Stage Partial (CSP) Dark modules (1 × 64, 2 × 128, 8 × 256, 8 × 512, and 4 × 1024 channels) that progressively analyze facial expressions. The initial 1xCSPDark layer-64 channel identifies local facial muscle activity and recognizes minor dynamics such as lowering the brow (AU4), which can be an early sign of the onset of pain. Subsequent 2xCSPDark layer-128 channel identifies regional patterns such as eye or nose area tension. They are then processed by the network using dual 8xCSPDark layers (256 and 512 channels), where the 256-channel layer recognizes partial orbital tightening (AU6), while the 512-channel layer integrates multiple AUs characteristics of severe pain grimaces. Finally, the 4xCSPDark layer-1024 channels synthesize these inputs into a global facial context, enabling precise mapping to the PSPI scores. The YOLOv4’s neck architecture employs multi-scale feature fusion to enhance detection sensitivity across diverse patient physiognomies. This design enables the detection of the full spectrum of pain indicators—from subtle brow lowering (AU4) to pronounced orbital tightening (AU6/AU7). The processed features are then passed to the head network, which generates three outputs: bounding box, pain intensity classification, and detection confidence.

(2) maxbatches=numberofclasses∗2000

### 2.4. Model Optimization

The number of network levels directly determines the ability to extract features. Our implementation employed an optimized layer freezing technique to preserve pretrained knowledge while specializing for chest pain detection. This method improves transfer learning efficiency by keeping important pretrained weights intact. Lower layers (1–128) remain frozen to maintain robust extraction of universal basic facial features, which form the foundation for AUs analysis. Mid layers (129–137) are partially fine-tuned to adapt to regional pain expression, while fully fine-tuning deeper layers (138–159) specialize to recognize complex pain-specific AU configurations. The custom models were trained with 10,000 max_batches, and the value of the learning rate was set to 0.01.

The YOLOv4 model was optimized to reduce overfitting and speed improvement. The choice of training cycles and learning rates in different scenarios is based on optimizing model performance, convergence speed, and generalization. The strategy varies between maskless and masked cases due to differences in feature extraction complexity. For the maskless cases, the YOLOv4 model resulted in two scenarios regarding optimization approaches: a 30,000 cycle training time and a learning rate of 0.005, and a 60,000 cycle training time and a learning rate of 0.001. The two scenarios regarding optimization approaches in the mask cases included a 20,000 cycle training time and a learning rate of 0.005 and a 50,000 cycle training time and a learning rate of 0.001.

Even though the custom YOLOv4 model was initially chosen, we could not ensure that the model trained in this version of the YOLOv4 would have the best performance. This study also evaluates the different types of YOLOv5, YOLOv7, and YOLOv8 model architectures for their capability to perform pain management tasks in real time. Reducing the occurrence of misjudgments, the accuracy perspective was prioritized. YOLO’s neural architecture type x was chosen because of its high accuracy among other YOLO neural architecture families. The base neural network architectures of YOLOv5x and YOLOv8x were configured with YOLOv5x.yaml and YOLOv8x.yaml, respectively. The experiment’s configuration consists of the yaml file, and learning rate hyperparameters were setted at 0.01 and 0.1, respectively. The setting of the YOLOv5x and YOLOv8x batch_size was 16, and the img_size was set at 480 with 200 epochs. Given that our data level size tends to be small- and medium-scaled, we still kept the best weight of the pretrained YOLOv7x model. The hyperparameter configuration of YOLOv7x was similar to YOLOv5x’s configuration. However, YOLOv7x.yaml was based on a neural network architecture, and the batch_size was set at 14. Even though YOLOv8x had the fastest training time, a PILASFE required high recall with stable accuracy. Therefore, YOLOv5x was selected. All of the models were trained with the following distribution of data: 70% data training, 20% data validation, and 10% data testing.

### 2.5. Model Evaluation

The model’s performances were evaluated using detection accuracy metrics such as mAP (mean Average Precision), precision (P), recall (R), F1-score, and computational efficiency under two scenarios, masked and maskless conditions, to assess its viability for clinical deployment in chest pain assessment.

### 2.6. Pain Management Implementation

Based on the evaluation metrics of all models, the potential models for the PILASFE foundation were the YOLOv5x model and the custom YOLOv4 model. However, YOLOv5x had difficulty analyzing facial expressions in the face mask scenario. This circumstance strengthens the custom YOLOv4 model that was able to be chosen for the PILASFE. In determining the pain intensity level, the hardware environment can process an average of 20 image recognition frames per second when performing 1280 × 720 image recognition. Each frame of the video was analyzed, and if a face was detected, it was classified into one of the pain intensity levels. The detected class is stored in a recognition class as a cumulative list. A single misclassified frame might cause incorrect pain detection. Therefore, the system waits for at least 16 frames as a threshold to determine stable classification in a sliding window. Every time a pain level was confirmed by passing the threshold, the increment process occurred in the counter variable. After processing the frames, the system calculated the percentages for each category using the counter variable. The pain intensity classification was determined by counting the occurrences of detected pain levels within a rolling window of the results of face expression recognition.

To provide a quick medical response and appropriate medical action, colors were used as user-centric features to visualize the patient’s level of pain intensity. Red indicates severe pain, orange represents slight pain, and green represents no pain. The ambiguity of facial expressions regarding the representation of pain levels is reflected in the color gray, which represents an uncategorized pain level. According to these color codes, the message displayed for pain’s categorization includes “not sure” (color = gray), “good” (color = green), “pain” (color = orange), and “alert” (color = red) as shown in Table 4.

In terms of deploying the model for real-time pain monitoring, a web-based interface was developed to visualize pain levels with color-coded alerts. Equipping the streaming input for the detection task, the Larix broadcaster software version 1.3.12 was first installed on the smartphone. The medium lens on the smartphone captured the object as the input data. The setting connection of the Larix broadcaster software was adjusted with an IP address of the Smart Campus Identification System server developed by Tunghai University. WiFi-5/6 was employed to transmit stream image data in a stable setting. Moreover, FFmpeg was used to convert the image data to RTMP format and upload it to the SRS cloud. The web page obtained images by connecting to the server’s IP address and then used the proposed model and transmitted the recognition results. Finally, the result of PILASFE appeared on the web page utilized by the Flask gateway interface to the web server. This enables real-time monitoring, early intervention, and improved emergency care efficiency.

## 3. Results

This study employed the YOLOv4, YOLOv5x, YOLOv7x, and YOLOv8x models. Table 5 summarizes the training results of YOLOv4 models in the detection of facial expression as an indication of chest pain intensity. The YOLOv4 model acquired an inference speed of 87.1 FPS and required 5.8 h of training time. On the other hand, YOLOv4t achieved a significant increase in inference speed (152.4 FPS) and reduced training time duration (4.3 h), with the cost of a 4.4% reduction in detection accuracy.

**Figure 3:**
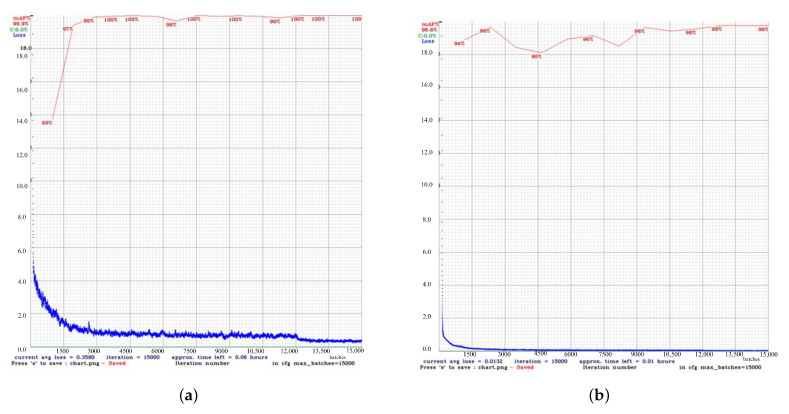
Performancecomparison between the custom YOLOv4 and YOLOv4t models in maskless detection tasks. (**a**) The results of the custom YOLOv4 model. (**b**) The results of the YOLOv4t model.

Figure 3 shows the results of the comparison of the performance of YOLOv4 and YOLOv4t in maskless settings. The YOLOv4 model proved to be more accurate and training-stable, with 99% mAP after around 2500 iterations (Figure 3a). For comparison, the dynamics of YOLOv4t training were less predictable, but at iteration 2,500, YOLOv4t had a competitive mAP of 98% (Figure 3b).

**Figure 4:**
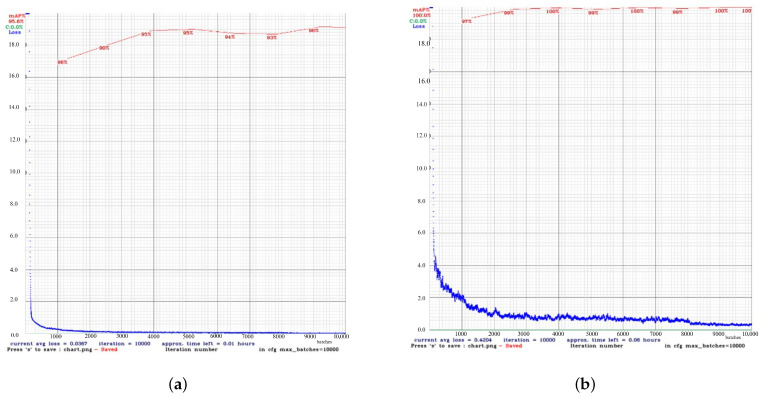
Performance comparison between the custom YOLOv4 and YOLOv4t models in masked detection tasks. (**a**) The results of the custom YOLOv4 model. (**b**) The results of the YOLOv4t model.

For masked scenarios (Figure 4), the learning process gradually occurred in both models, although with a certain degree of difference. YOLOv4t achieved a 4.4% increase in mAP compared to YOLOv4. However, the final mean loss (0, 4204) of YOLOv4t was considerably higher than that of YOLOv4.

The best overall performance was achieved by YOLOv4 with 128 network layers. With these layers, YOLOv4 had the fastest training time at 6.55 h with an accuracy of 100% mAP@50, as seen in Table 6. In contrast, YOLOv4 with 159 network layers showed the lowest training loss (0.06) and relatively worse localization results.

Table 7 presents the performance of a custom YOLOv4 model trained in pain management, comparing the results between models with and without masks. In both cases, the proposed model achieved an accuracy of up to 98%. Due to the simplified feature extraction that focuses on the eyes and forehead rather than the entire face, the training time with masks was faster than that of training without masks.

The training results of the YOLOv5x, YOLOv7x, and YOLOv8x models, which are written in PyTorch version 1.8, are shown in Table 8. It shows the comparison between the performances of YOLOv5x, YOLOv7x, and YOLOv8x in different performance metrics. In general, the models achieved an effectiveness rate of more than 90%. YOLOv5x not only obtained the best recall at 99.9% but also achieved the highest precision with 99.8% as well as mAP@50 and mAP@50–95. However, YOLOv5x’s training time was longer than YOLOv8x’s. Based on these results, YOLOv5x can be considered as a potential model if stable accuracy and the highest recalls are the priorities. In contrast to both YOLOv5x and YOLOv8x’s mAP@50–95, YOLOv7x performed the worst among them as it had difficulties with the precise localization of the bounding box. YOLOv7x also had weaknesses in recall and training time. The performance of YOLOv8x was significantly different from that of YOLOv7x. The strength of YOLOv8x was in training time, precision, and the mAP@50–95. The fastest training time and best overall performance were achieved by the YOLOv8x. Compared to the YOLOv5x and YOLOv7x models, it had the shortest training time at 12.489 h, had fewer false positives, and was able to generalize better across different IoU thresholds, making the YOLOv8x the most efficient model.

**Figure 5:**
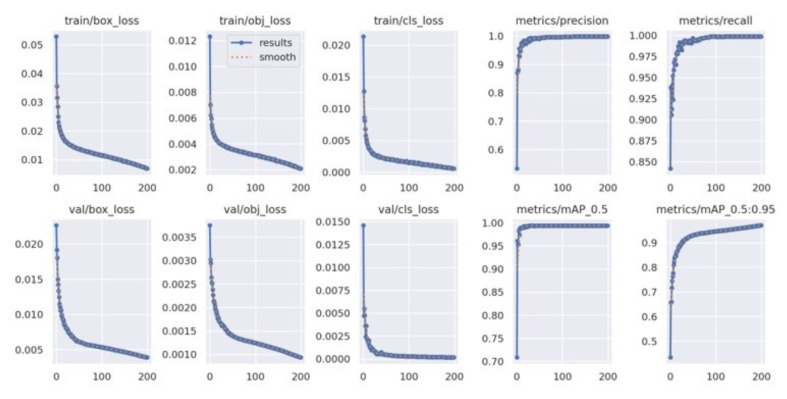
YOLOv5x’s train loss curve.

Figure 5 presents the training curves of the YOLOv5x model during its learning period over 200 epochs. During training, the model was able to minimize errors. In the bounding box loss plot, the curve decreased over time, demonstrating that the model is improving at localizing objects. The object loss plot shows the gradually decreasing curve, meaning that the model is improving in distinguishing objects from the background. The trends of the validation loss curves were similar to the trends of the training box loss curves.

**Figure 6:**
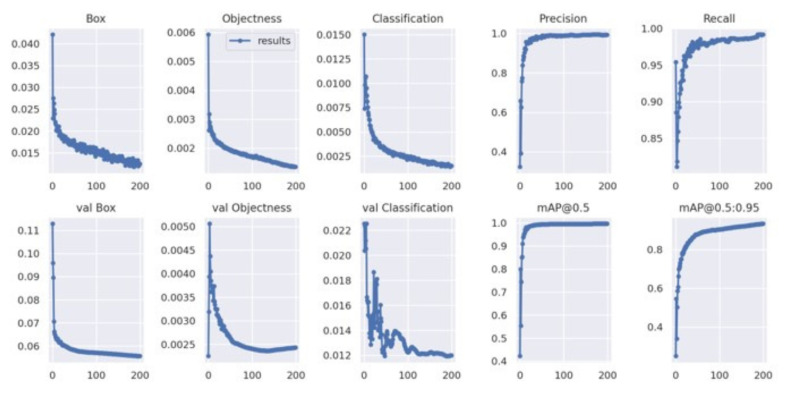
YOLOv7’s train loss curve.

The learning process of YOLOv7x across 200 epochs is shown in Figure 6. The training box loss plot shows the gradual decreasing curve, outlining the stable model learning and improved bounding box localization over epochs. The classification loss plot depicts the starts with significant increases and drops, pointing to better classification of different pain intensity levels. The validation classification loss plot shows some fluctuations around 50–100 epochs; hence, the model experienced learning instability.

**Figure 7:**
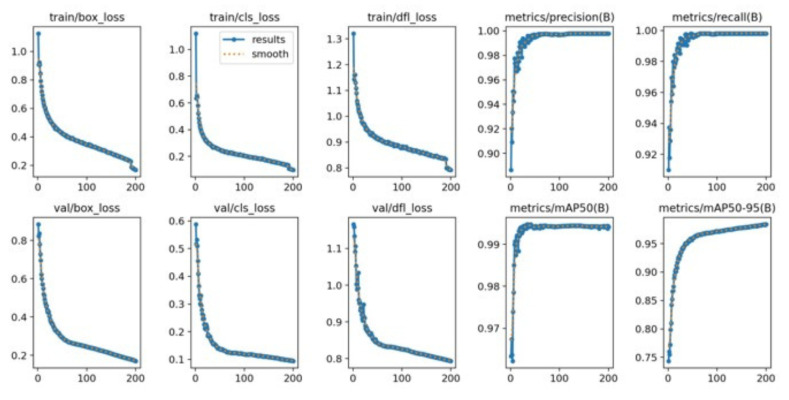
YOLOv8’s train loss curve.

Figure 7 shows the training loss and performance metric curves for the YOLOv8x model. The training bounding box loss plot decreases steadily, showing a similar trend to the training classification loss plot. The validation bounding box loss plot and the validation classification loss plot exhibit a downward trend. The mAP@50–95 increased rapidly during training, and both precision and recall remained high throughout the process.

**Figure 8:**
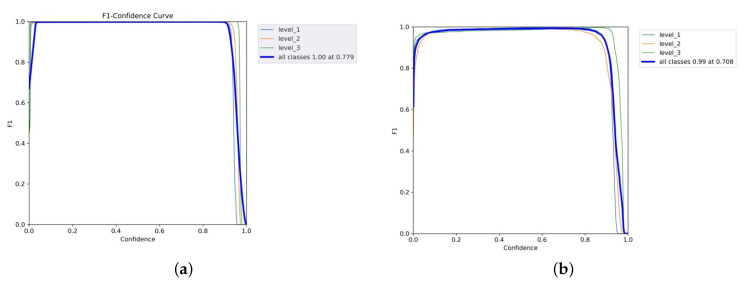
F1–confidence curves of YOLOv5x and YOLOv7x models. (**a**) YOLOv5x’s F1–confidence curve. (**b**) YOLOv7x’s F1–confidence curve.

The confidence curve is employed to evaluate the reliability model. Figure 8a shows that the F1-score of all classes of the YOLOv5x was considerable, meaning that it performed well. The F1-score of every class also rose by 1.0 as the confidence value was 0.779, which means that this is the value of confidence where the level of false positives and false negatives will reduce accordingly. The YOLOv5x model seems to be able to classify various levels of pain with minimal error. In the meantime, the F1-score of the YOLOv7x was optimal (0.99) when the confidence was 0.708, as illustrated in Figure 8b. Individual classes maintained a high F1-score across a wide range of confidence thresholds. The model performs consistently well for all levels of intensity pain with only slight variations between classes. The broader plateau indicates strong classification performance; therefore, the model is not overly sensitive to alternations in the levels of confidence. Thus, YOLOv7x achieves adequate optimization under the primary need of robustness and adaptivity to threshold alterations with minimal gain at some intensity.

**Figure 9:**
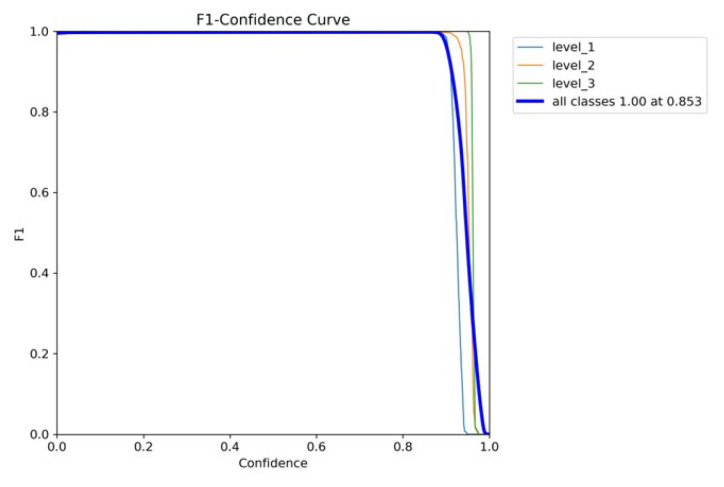
YOLOv8’s F1–confidence curve.

The high performance of YOLOv8x is shown in Figure 9. Its macro F1-score was 1.00, and its confidence limit was 0.853. Individual classes also demonstrate high values of F1-scores in YOLOv8x; however, their plot is smoother than in the case of YOLOv7x. The model gives the best-balanced accuracy (F1-score = 0.853) in the precision–recall tradeoff, implying that the model works best when both precision and recall are powerfully considered. In contrast, non-optimal tradeoffs can produce sharp tradeoffs against recall, with the risk that clinical utility on sensitive tasks such as detecting chest pain would be at risk. YOLOv8x demonstrates high-quality detection capabilities.

**Figure 10:**
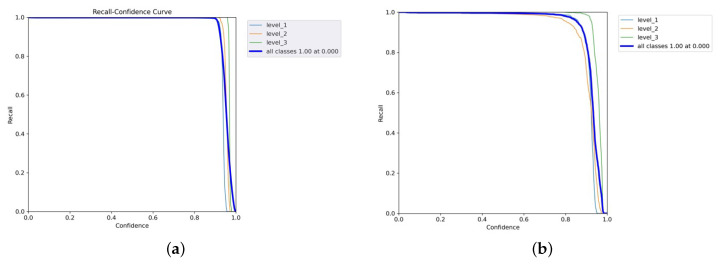
Recall–confidence curves of YOLOv5x and YOLOv7x models. (**a**) YOLOv5x’s recall–confidence curve. (**b**) YOLOv7x’s recall–confidence curve.

**Figure 11:**
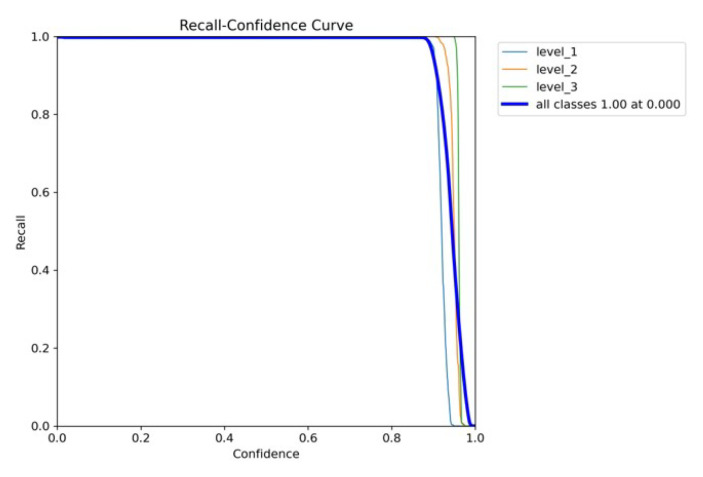
YOLOv8’s recall–confidence curve.

Figure 10a,b and Figure 11 are the recall–confidence curves of YOLOv5x, YOLOv7x, and YOLOv8x that depict how recall is affected by the confidence threshold. It ensures that no pain cases are missed, especially in medical settings where missing pain is unacceptable.

## 4. Discussion

The comparative evaluation of YOLOv4 and YOLOv4t highlights the trade-off between accuracy and computational efficiency. Although YOLOv4t is significantly faster and requires less training time, this advantage comes at the expense of reduced accuracy and less stable learning dynamics. In maskless settings, YOLOv4’s loss gradually decreased to 0.35, which means that subtle facial expressions related to different intensities of pain in the chest have been successfully learned. This stability is essential in clinical contexts where diagnostic precision is critical—particularly when identifying subtle pain-related facial AUs like brow lowering (AU4) or orbital tightening (AU6/AU7). In contrast, YOLOv4t reached a lower loss at approximately 0.13, indicating a lower error rate. Although efficient and faster, YOLOv4t may be less stable and prone to fluctuations compared to YOLOv4, potentially impacting detection accuracy for complex facial expressions. In masked scenarios, YOLOv4t achieved higher mAP yet exhibited considerably higher loss, indicating overfitting or impaired generality. In summary, while YOLOv4t offers faster performance, the consistently higher accuracy and stability of YOLOv4 make it better suited for detailed and reliable detection of chest pain levels, where diagnostics precision outweighs computational efficiency.

Regarding model optimization, the hierarchical layer structure of the YOLOv4 architecture enables it to perform the task of feature extraction more effectively. Consecutive layers within the network capture details of facial expression patterns that are increasingly related to pain assessment. Notably, the 128-layer YOLOv4 architecture provides the best balance between computational speed and clinical precision, achieving robust performance in pain intensity classification. In contrast, the 159-layer YOLOv4 version yielded the lowest training loss but produced worse localization results, implying that although deep models give stable converging learning, they can cause poor spatial localization of pain-related facial AUs. Based on these results, the 128-layer YOLOv4 configuration offers the most advantageous solution for balancing accuracy, stability, and localization in clinical settings.

The training process and model’s convergence are influenced by the learning rate and max batch size. As the key hyperparameters in the optimization model, the learning rate value was reduced and the maximum batch size value was gradually increased. Hyperparameter tuning was experienced with two configurations: a 30,000 cycle training time at a learning rate of 0.005 and a 60,000 cycle training time at a learning rate of 0.001. For the maskless optimization model, the results indicate diminishing returns. Specifically, extending training from 30,000 to 60,000 cycles yielded only a marginal reduction of 0.0005 in the loss value and no improvement in the mAP, which remained at 99.97%. Not only is the maskless model experienced in optimization strategies, but the mask model also undergoes optimization. In the masked optimization experiments, two training configurations were evaluated: 20,000 cycle training time with a learning rate of 0.005 and 50,000 cycles with a learning rate of 0.001. The results demonstrate that both settings achieved strong convergence, as reflected by stable loss curves and consistently high mAP values. The final average loss of 0.2138 indicates that the model was well-trained with improved prediction accuracy. These findings underscore that while hyperparameter optimization effectively enhances model performance, prolonged training beyond a certain point provides minimal gains and increases computational cost. Overall, the results highlight the importance of balancing computational efficiency and model precision, particularly in clinical applications where reliable yet timely diagnostic support is essential. The performances of both the maskless and masked optimization model are provided in the Supplementary File.

The evaluation of PyTorch-based models provides additional evidence supporting the observations, particularly regarding the balance between accuracy, stability, and computational efficiency. YOLOv5x achieved the best recall, indicating that the YOLOv5x model detects almost all objects. The training behavior of the YOLOv5x model indicates that it was able to minimize errors effectively over 200 epochs. Overall, the model is well trained and highly accurate and generalizes well without overfitting. These findings highlight that YOLOv5x is a potential model for pain-related facial expression detection.

**Figure 12:**
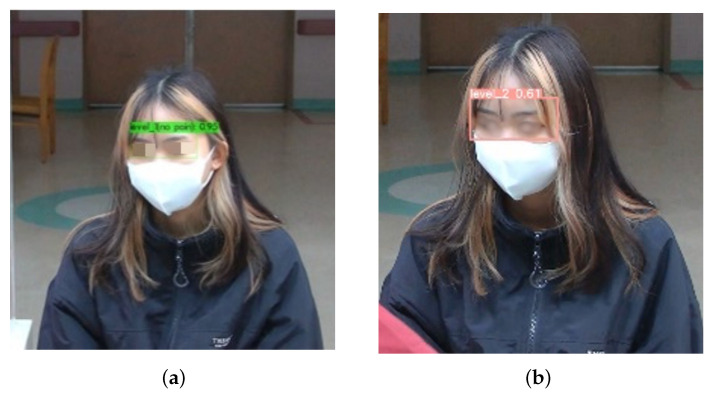
Performances of YOLO series model with mask. (**a**) YOLOv4; (**b**) YOLOv5.

Figure 12 shows the comparison of the detection performance of the custom YOLOv4 and YOLOv5x models in the masked condition. YOLOv4 did not detect pain with a high confidence score of 0.96. As such, the model is reliable in this scenario due to its ability to effectively capture facial features despite the masks. In contrast, YOLOv5x had reduced confidence, possibly due to difficulties in interpreting expressions with a mask. YOLOv7x and YOLOv8x completely failed to detect pain intensity, highlighting the potential weaknesses in their feature extraction for masked individuals. YOLOv7x and YOLOv8x may lack fine-tuning, thus leading to poor generalization. YOLOv7x and YOLOv8x may prioritize high-precision detection, discarding ambiguous or low-confidence detection. Therefore, YOLOv4 is the most effective model in detecting the intensity of chest pain.

**Figure 13:**
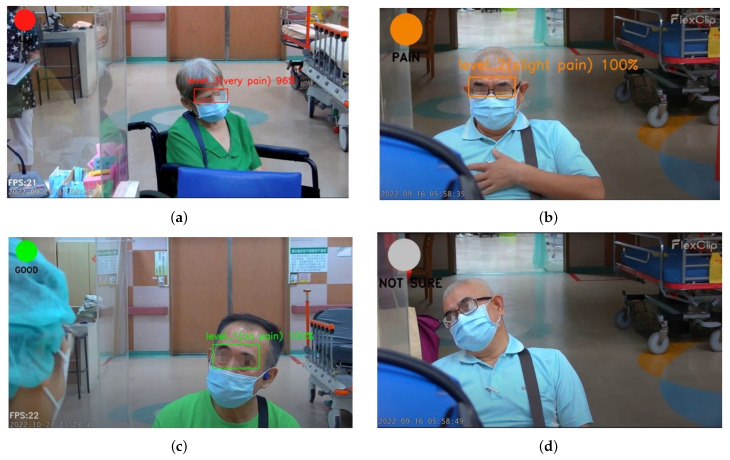
Pain intensity level notification. (**a**) Level 3. (**b**) Level 2. (**c**) Level 1. (**d**) Uncategorized.

Figure 13a–d show the effectiveness of the custom YOLOv4 model for classification of pain intensity based on facial expressions. The model performs well despite masks. The system accommodated the uncertain intensity of pain that is shown in Figure 13d, indicating insufficient facial expression cues.

Facial data are very sensitive, and therefore, it is necessary to handle data properly, encrypt data, and adhere to privacy policies. Despite the high performance of the proposed model in the study dataset, the training data in this study are not comprehensive with regard to the full heterogeneity of global population in terms of age, gender, and ethnicity. This limitation may constrain the system’s fairness and generalizability across a broader patient population, which increases the potential risk of bias. There is also a risk of misdiagnosis in cases where the system either overestimates or underestimates pain levels. Another shortcoming is the lack of clinical metadata, including symptoms and comorbidities, which limits the system to drawing inferences about the etiological causes or severity of chest pain, such as myocardial infarction. To address this, we emphasize that the proposed system is intended as a decision support tool rather than a replacement for clinical judgment. Importantly, it is not designed for triage or diagnostic purposes, and healthcare professionals will still have the responsibility to make decisions when it comes to patient care.

Although several studies have investigated automated pain assessment, there remains a notable lack of research focused specifically on addressing the recognition of chest pain intensity in real time. An accuracy of 94.95% was reported by Chavan [21], who employed the hybrid optimization-based deep CNN, while Alghamdi [36] achieved 98.93% accuracy with sparse autoencoders under a 10-fold cross validation scheme. Hausmann [23] employed the YOLOv6 model to determine pain in post-surgical neonates, with the model achieving a precision of 63.2%. A previous study [37] proposed a hybrid convolutional–transformer approach, which obtained a mean square error of 0.2094. Compared with these approaches, the proposed approach demonstrates competitive performance, as summarized in Table 9.

## 5. Conclusions and Future Work

The proposed system demonstrates the potential of automated pain assessment based on facial expressions using a custom YOLOv4 model fine-tuned with PSPI scores, achieving 97% precision with the fastest training time. The system enables easy and flexible installation on laptops and smartphones through the inclusion of real-time streaming and a web-based interface with color-coded indicators, which is particularly relevant in the context of emergency care settings when the number of human resources is limited. Importantly, the PILASFE platform lacks the element of diagnostic information on the etiology or severity of chest pain and is therefore not to be used as a triage tool; instead, it is intended as a decision support system to complement, rather than replace, clinical judgment and to support patient monitoring. For future work, the system will be enhanced within an edge computing schema using a lightweight model to optimize performance for real-time assessments. Furthermore, it will be integrated with multimodal health records to strengthen its robustness and potential applicability in the healthcare setting.

## Figures and Tables

**Table 1:** Number of studies on pain intensity.

Authors	Model	Result	Dataset	Intensity Pain Level
Chavan [21]	Hybrid optimization deep CNN	Accuracy 94.95%	UNBC-McMaster Shoulder Pain Archive dataset	-
Ismail [22]	ResNet18	Accuracy 87.89%	UNBC-McMaster Shoulder Pain Archive dataset	6
Hausmann [23]	YOLOv6	Accuracy 62.7%	WIDER Face dataset, USF-MNPAD-I dataset, USF-MNPAD-II dataset	2
Gkikas [24]	Spatial module	Accuracy 39.77%	GGFace dataset, AffectNet, Compound Facial Expressions of Emotions Database, RAF Face Database basic, RAF Face Database compound, ECG Heartbeat Categorization dataset	6
Swetha [25]	Dense-CNN	Accuracy 75%	Self collection dataset	4
Wu [26]	Global and local attention-CNN	Accuracy 56.45%	UNBC-McMaster Shoulder Pain Archive dataset	4
Alghamdi [13]	Dual-CNN	Accuracy 99.10%	UNBC-McMaster Shoulder Pain Archive dataset	4

**Table 2:** Sample distribution based on pain level classification.

Pain Level	PSPI Score	Category	Number of Maskless Samples	Number of Masked Samples
Level 1	0	No pain	3960	4627
Level 2	1–6	Slight pain	3872	5627
Level 3	7–16	Severe pain	3889	5456

**Table 3:** Parameters.

Parameters	Values
batch	64
subdivisions	64
width	736
height	448
max batches	6000
steps	4800, 5400
filters	24

**Table 4:** Pain severity notification.

Categorized	Message	Color
uncategorized	not sure	gray
no pain	good	green
slight pain	pain	orange
severe pain	alert	red

**Table 5:** The training results of YOLOv4.

Models	mAP@50	FPS	Training Time (Hours)
YOLOv4	100%	87.1	5.8
YOLOv4t	95.6%	239.5	1.5

**Table 6:** Training results of pain classification model with YOLOv4.

Number of Network Layers	mAP@0.50	Average IOU	Average Loss	Precision	Training Time (Hours)
128	100%	84.64%	0.09	0.97	6.55
137	99.94%	84.64%	0.07	0.97	7.15
152	99.96%	83.64%	0.08	0.96	7.03
159	99.94%	84.64%	0.06	0.97	7.23

**Table 7:** Custom model results with optimization.

Scenario	mAP@0.5	AVG Loss	Total Training Time (Hours)
Without Mask	99%	0.20	30
With Mask	99.9%	0.14	28

**Table 8:** The training results of YOLOv5x, YOLOv7x, and YOLOv8x with PyTorch framework.

Models	Precision	Recall	mAP@50	mAP@50–95	Training Time (Hours)
YOLOv5x	99.8%	99.9%	99.4%	97.1%	14.643
YOLOv7x	99.4%	99.2%	99.7%	93.4%	20.308
YOLOv8x	99.8%	99.8%	99.4%	98.4%	12.489

**Table 9:** Comparative performance of the proposed model with existing works in detecting pain intensity based on facial expression.

Authors	Model	Result
Chavan [21]	Hybrid optimization-based deep CNN	Accuracy 94.95%
Alghamdi [36]	Sparse autoencoders	Accuracy 98.93%
Hausmann [23]	YOLOv6	Precision 63.2%
Mao [37]	Hybrid conv-transformer	Mean square error 0.2094
Proposed Model	YOLOv4custom	Precision 97%

## Data Availability

The data are not publicly available due to ethical restrictions.

## Supplementary Materials

The following supporting information can be downloaded at https://www.mdpi.com/article/10.3390/diagnostics15202661/s1, Figure S1: Proposed model pseudocode; Figure S2: Maskless optimization model performance with 30,000 cycle time; Figure S3: Maskless optimization model performance with 60,000 cycle time; Figure S4: Mask optimization model performance with 20,000 cycle time; Figure S5: Mask optimization model performance with 50,000 cycle time; Figure S6a.YOLOv5x’s precision–confidence curve; Figure S6b. YOLOv7x’s precision–confidence curve; Figure S7. YOLOv8x’s precision–confidence curve.

## Abbreviations

AI	artificial intelligence
AU	action unit
CSP	cross-stage partial
CNN	convolutional neural network
CVD	cardiovascular disease
ER	emergency care
FACS	facial action coding system
FPS	frames per second
mAP	mean average precision
NCD	non-communicable disease
P	precision
PSPI	Prkachin and Solomon Pain Intensity
R	recall
SSD	single-stage detector
YOLO	You Only Look Once
