# Supplementary material for: A Public Health Approach to Automated Pain Intensity Recognition in Chest Pain Patients via Facial Expression Analysis for Emergency Care Prioritization
Source: Diagnostics (Basel). 2025 Oct 21;15(20):2661. doi: 10.3390/diagnostics15202661 (PMC12564096; doi:10.3390/diagnostics15202661)
Supplement: Supplementary file 1 [file diagnostics-15-02661-s001.zip › diagnostics-3834619-supplementary.pdf]

Supplementary Material

"A Public Health Approach to Automated Pain Intensity Recognition in Chest Pain Patients via Facial Expression Analysis for Emergency Care Prioritization"

```
1.Initialization
2.Input sources video stream RTMP or local file
3.Pre-trained YOLOv4 model (chestpain_best128.weights, chestpain.cfg) with confidence threshold=0.4, NMS threshold=0.4, buffer frames=20, minimum detection frames for decision=16
4.Object detection module:
Input size: batch=64, subdivisions=64, width=1280, height=704, channels=3, momentum=0.949
Function backbone= conv 32, (1xCSPDark, 64), (2xCSPDark, 128), (8xCSPDark, 256), (8xCSPDark, 512), (4xCSPDark,1024)
Function neck= YOLOv4-SPPNet
Function head= (P3, P4, P5, 3)

5.WHILE video_stream.is_open():
  READ frame FROM video_stream

  IF perform_detection == True:
    CONVERT frame TO Darknet format
    RUN YOLOv4 detection ON frame
        EXTRACT (label, confidence, bbox) FROM detections

    IF detections EXIST:
      SELECT detection WITH highest confidence
        DRAW bounding box WITH color BASED ON label:
          - Green: level_1(no pain)
          - Orange: level_2(slight pain)
          - Red: level_3(very pain)

      UPDATE rolling buffer (last 20 frames):
        ADD current label TO recognition_classes
        IF buffer FULL (20 frames):
          REMOVE oldest entry
      CALCULATE label counts IN buffer:
        CASE 1: >16 "no pain" frames → Good
        CASE 2: >16 "slight pain" frames → Pain
        CASE 3: >16 "very pain" frames → Alert
        CASE 4: Mixed pain levels → Use majority vote
        DEFAULT: 'no detection' (Not Sure)

      UPDATE class_occurrences counter
      DISPLAY status circle (Green/Orange/Red)
    ELSE:
      COUNT AS 'no detection'

    STREAM annotated frame TO web interface

  ELSE: # Detection paused
    STREAM original frame

6.  Decision logic:
IF total_detected_frames > 0 AND noise_ratio(<40% undetected):
  CALCULATE percentages:
      percent_no_pain= (count_no_pain / total_detected) * 100
          percent_slight_pain = (count_slight_pain / total_detected) * 100
          percent_severe_pain= (count_severe_pain / total_detected) * 100
      RETURN formatted string: "percent_no_pain: X%, percent_slight_pain: Y%, percent_severe_pain: Z%"
ELSE:
  RETURN "Insufficient detections"

7.For consistent processing use resize frames to 854x480
8.Maintain 20-frame buffer for stable decisions
9.Error-handling: auto reset counters when detection is toggled off
10.Noise filtering (ignore if > 40% frames undetected)
```

Figure S1. Proposed model pseudocode

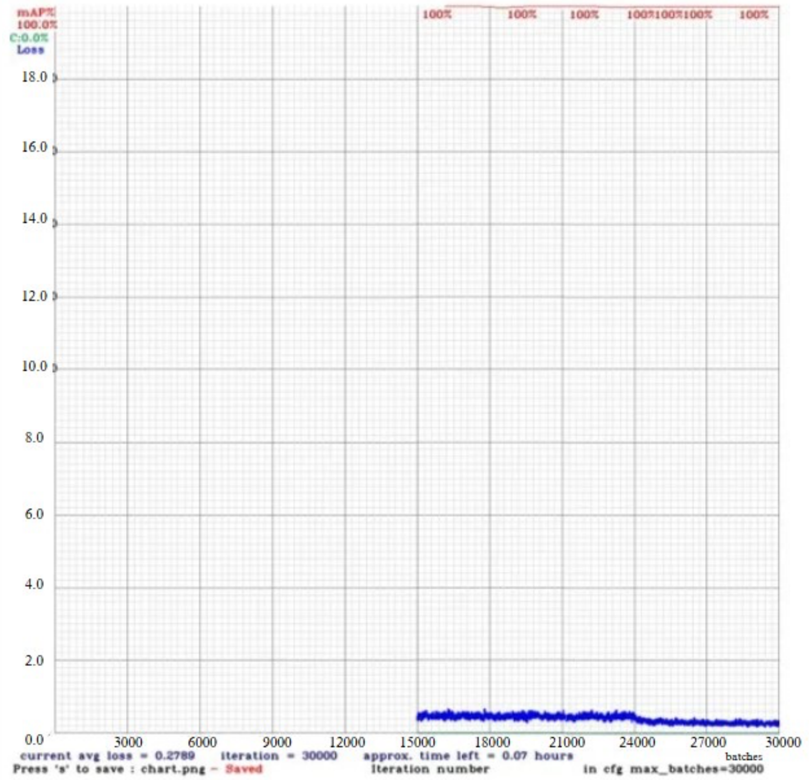

Figure S2. Maskless optimization model performance with 30,000 cycle time

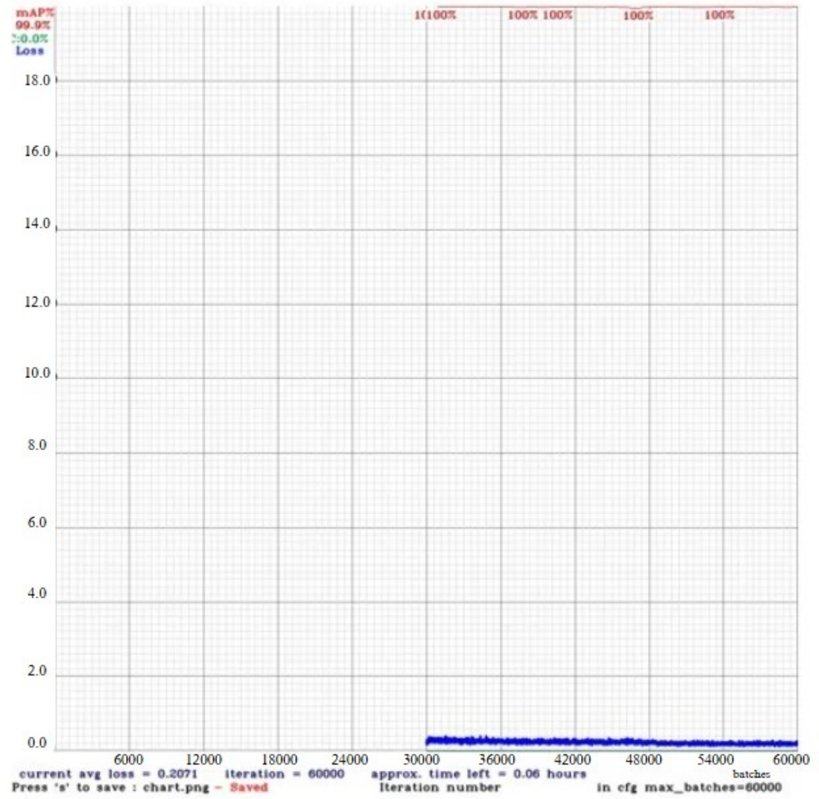

Figure S3. Maskless optimization model performance with 60,000 cycle time

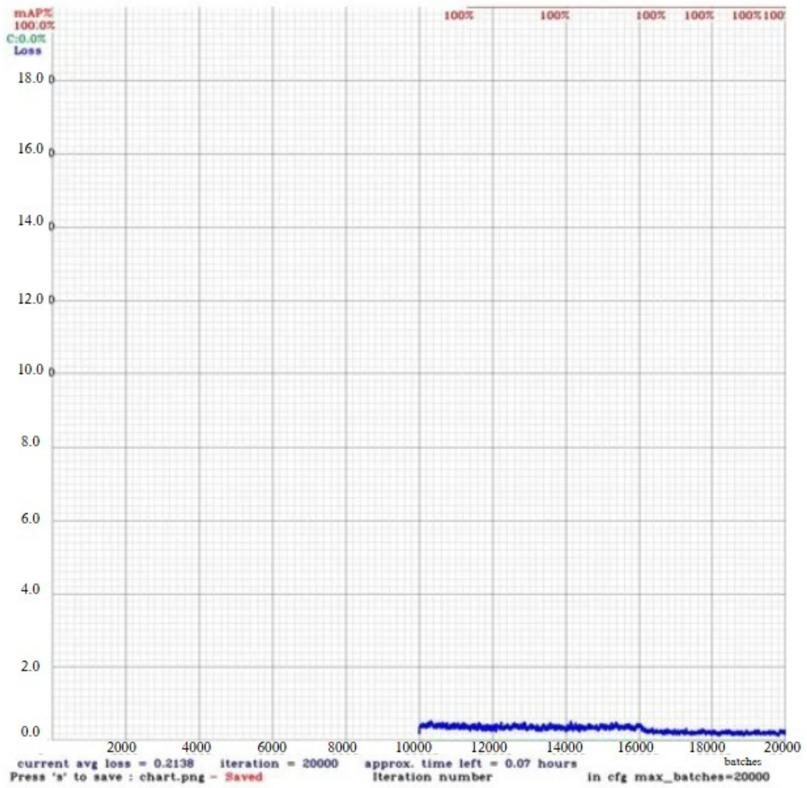

Figure S4. Mask optimization model performance with 20,000 cycle time

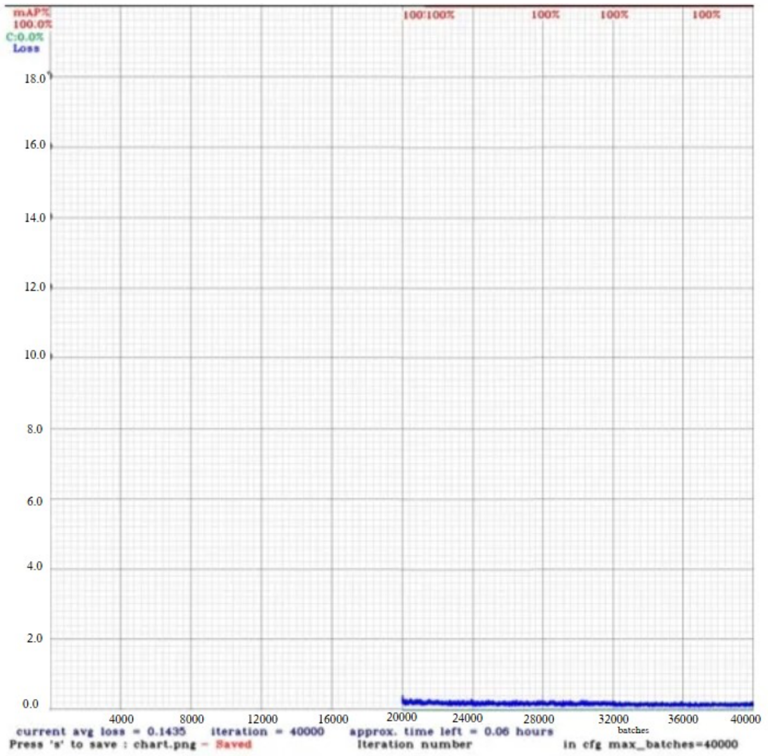

Figure S5. Mask optimization model performance with 50,000 cycle time.

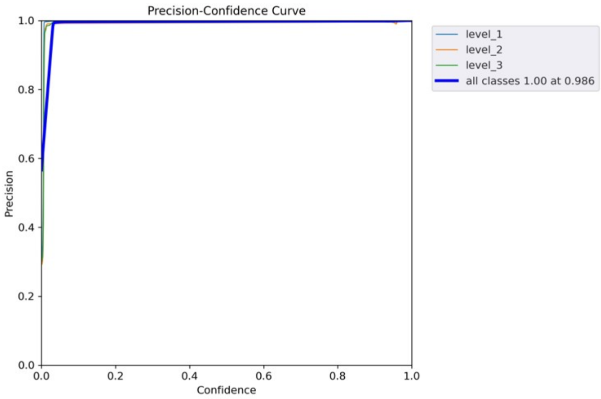

a. YOLOv5x's precision-confidence curve.

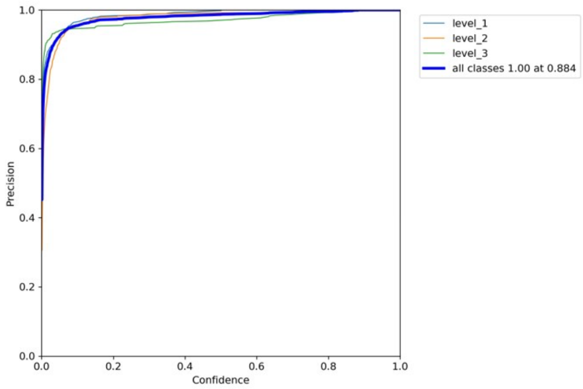

b. YOLOv7x's precision-confidence curve.

Figure S6. Precision-confidence curves of YOLOv5x and YOLOv7x models.

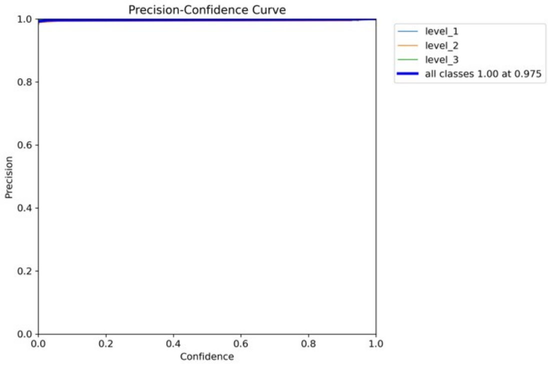

Figure S7. YOLOv8x’s precision-confidence curve.
